# EAAT5 Glutamate Transporter-Mediated Inhibition in the Vertebrate Retina

**DOI:** 10.3389/fncel.2021.662859

**Published:** 2021-05-06

**Authors:** Peter D. Lukasiewcz, Gregory W. Bligard, James D. DeBrecht

**Affiliations:** ^1^Department of Ophthalmology & Visual Sciences, Washington University School of Medicine in St. Louis, St. Louis, MO, United States; ^2^Department of Neuroscience, Washington University School of Medicine in St. Louis, St. Louis, MO, United States

**Keywords:** EAAT5, glutamate, transporter, retina, inhibition, light-response

## Abstract

Glutamate transporters typically remove glutamate from the synaptic cleft. In addition, all glutamate transporters have a chloride channel, which is opened upon glutamate binding to the transporter. There are five types of glutamate transporter (EAATs 1–5, excitatory amino acid transporters), which have distinct chloride conductances. Some EAATs that have low chloride conductances, remove glutamate from the synaptic cleft most effectively (e.g., EAAT1). By contrast, EAATs that have high chloride conductances, remove glutamate less effectively (e.g., EAAT5). We have studied EAAT5 in the retina. In the retina, light activates a chloride current, mediated by the glutamate activation of EAAT5. EAAT5 is not a significant contributor to lateral inhibition in the retina. Instead, it is the main source of autoinhibition to rod bipolar cells (RBCs). EAAT5-mediated inhibition has a substantial effect on synaptic transmission from RBCs to downstream retinal neurons.

## Introduction

Glutamate is not enzymatically broken down. Thus, the uptake of glutamate from the synapse terminates the glutamate signal. Typically, the glutamate signaling ends by the rapid diffusion of glutamate from the synapse. Subsequent glutamate reuptake into glia and neurons clears released glutamate from the synapse, ultimately terminating the glutamate-evoked, excitatory signal. Generally, glial glutamate transporters play a larger role than neuronal glutamate transporters in clearing glutamate from the synapse and terminating the excitatory signal in retina and the brain (Higgs and Lukasiewicz, 1999; Amara and Fontana, 2002).

**Figure 1:**
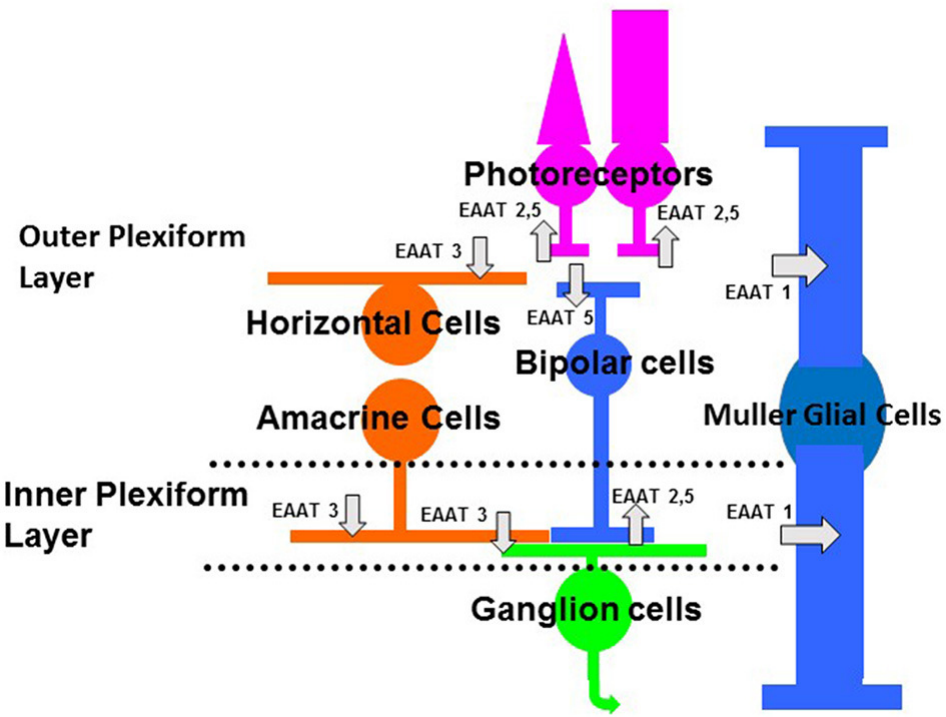
Schematic of the vertebrate retina showing the main classes of neurons and glia. Photoreceptors (rods, on right, and cones, on left) provide input to bipolar cells and horizontal cells. Synaptic contacts between photoreceptor and their post-synaptic targets occur in the outer plexiform layer. Bipolar cells, which relay information between photoreceptors and ganglion cells, provide input to amacrine cells and ganglion cells. Horizontal cells and amacrine cells are laterally ranging cells that modulate the flow of visual information in the outer and inner plexiform layers, respectively. The Muller cell is the main glial cell in the retina, spanning the entire retina thickness, from photoreceptors to ganglion cell axons. Glutamate transporters are indicated by the gray arrows. The subtypes of EAATs or glutamate transporters and the cell types on which they are located (see text for more explanation) are indicated in the schematic. Some glutamate transporters or EAATs are located on photoreceptors and bipolar cells, where they act as glutamate receptors that gate anion conductances (EAAT5) (Picaud et al., 1995b; Veruki et al., 2006). Glutamate transporters (EAAT1) are also on Muller cells, where they take up glutamate and terminate the excitatory signal (Higgs and Lukasiewicz, 1999).

There are five subtypes of EAATs 1–5, excitatory amino acid transporters (for reviews, see Amara and Fontana, 2002; Vandenberg and Ryan, 2013; Fahlke et al., 2016). Glutamate transporters are found on glia and neurons (see Figure 1). The isoforms, EAAT1–2 are primarily considered glial transporters and EAAT3–5 are primarily considered neuronal glutamate transporters (Amara and Fontana, 2002; Vandenberg and Ryan, 2013). There are exceptions to this general rule; EAAT2 is found on some retinal and brain neurons and EAAT5 is found on some retinal glia (Eliasof et al., 1998; Petr et al., 2015; Rimmele and Rosenberg, 2016).

All EAATs have two functions, they can bind and clear glutamate and they can function as anion channels (Fahlke et al., 2016). EAATs 1–3 clear glutamate from the synapse effectively but have relatively small anion conductances. EAATs 4 and 5 clear glutamate from the synapse relatively inefficiently (compared to EAATs 1–3), but they have large anion conductances (Fairman et al., 1995; Arriza et al., 1997; Fahlke et al., 2016). Machtens et al. (2015) describe the molecular mechanisms that form the anion conductance by EAATs.

This review focuses on the vertebrate retina. There are several isoforms of EAATs in the retina (Rauen, 2000). EAATs 1–3 are found on both glial cells and neurons and are efficient transporters of glutamate, but mediate a small chloride current (if at all). EAATs 1–3 are found in the retina. EAAT1 is primarily localized in the Muller glial cell (Rauen et al., 1996; Lehre et al., 1997; Pow and Barnett, 1999; Sarthy et al., 2005), as indicated in Figure 1. EAAT2 is found in photoreceptors and in bipolar cells (Rauen and Kanner, 1994; Eliasof et al., 1998), as shown in Figure 1. EAAT2 is also a presynaptic EAAT in the brain (Petr et al., 2015; Rimmele and Rosenberg, 2016). EAAT3 is found in the inner retina (Rauen et al., 1996; Schultz and Stell, 1996; Schniepp et al., 2004). There is EAAT3 immunocytochemical labeling of horizontal cells, amacrine cells, and ganglion cells (Figure 1). EAATs 4 and 5 mediate large chloride conductances (Fairman et al., 1995; Arriza et al., 1997), but only EAAT5 is found in the neurons and Muller cells of the retina (Arriza et al., 1997; Ward et al., 2004). EAAT4 immuno-labeling is not found in neurons or Muller glial cells of the retina (Ward et al., 2004). EAAT5, by contrast, mediates a large chloride current, is found almost exclusively in the retina (Arriza et al., 1997) and is the focus of this review (see Figure 1). Because glutamate binding to this isoform of EAAT gates a chloride conductance that mediates inhibition, EAAT5 affects visual processing in the retina, as noted below.

Numerous groups have studied EAAT5's role in visual processing at the photoreceptor and bipolar cell outputs (Eliasof and Werblin, 1993; Picaud et al., 1995b; Grant and Werblin, 1996; Palmer et al., 2003; Rabl et al., 2003; Veruki et al., 2006; Wersinger et al., 2006; Ichinose and Lukasiewicz, 2012; Bligard et al., 2020). EAAT4 is not found in the neurons or Muller glial cells of the retina, based on Northern blot experiments (Eliasof et al., 1998) or immunohistochemistry (Ward et al., 2004) and likely does not play a role in retinal processing. Thus, EAAT5, which mediates a large chloride conductance, is found almost exclusively in the retina and affects visual processing in the both synaptic layers of the retina, is the focus of this review.

## Cloning the EAAT5 Transporter

The EAATs were first cloned and expressed in oocytes by the Amara group (Amara and Fontana, 2002). The last EAAT cloned was EAAT5 by Arriza et al. (1997). This isoform was found to have a large chloride ion conductance associated with it. Northern blots showed that it was found primarily in retina (Arriza et al., 1997). Subsequent studies by Eliasof et al. (1998) showed the expression of EAATs in the salamander retina. EAAT5 immuno-signals were found in photoreceptors, Muller glial cells, and bipolar cells (Eliasof et al., 1998) (Figure 1). Functional studies, at the time, suggested that it has pre-synaptic and post-synaptic roles in photoreceptors and bipolar cells, respectively (Grant and Dowling, 1995; Picaud et al., 1995b; Grant and Werblin, 1996).

There is no EAAT5-specific antagonist or EAAT5 knockout animal. EAAT5 transporters' location and functions have been attributed to the use of EAAT5-specific antibodies (Eliasof et al., 1998; Wersinger et al., 2006). Additionally, EAAT5 was cloned from the human retina (Arriza et al., 1997), where it is found primarily by Northern blot analysis. EAAT5 also mediates a large chloride conductance, when heterologously expressed in frog oocytes (Arriza et al., 1997; Eliasof et al., 1998). Finally, the neurons or Muller glia of the retina do not express EAAT4, the other isoform with a large chloride conductance (Fairman et al., 1995). Thus, it is inferred that the large chloride conductance that is mediated by an EAAT is the EAAT5 transporter.

The roles of glutamate transporters in signaling have been well-studied in the retina (Picaud et al., 1995b; Grant and Werblin, 1996; Higgs and Lukasiewicz, 1999; Palmer et al., 2003; Wersinger et al., 2006; Ichinose and Lukasiewicz, 2012). In particular, variants of the glutamate transporters with large anion conductances have been investigated in retina. Typically, chloride mediates the anion current. Signaling in both synaptic layers, the outer plexiform layer, where photoreceptors make synaptic contacts with bipolar and horizontal cells, and the inner plexiform layer, where bipolar cells make synaptic contacts with amacrine and ganglion cells, EAATs shape the excitatory signal (Figure 1). Figure 1 shows a schematic view of EAATs found in the retina. EAAT5 is found in the outer plexiform layer on several neuronal types. It is found presynaptically on rod and cone photoreceptor terminals (Picaud et al., 1995a; Grant and Werblin, 1996). In addition, it is found post-synaptically on the dendrites of teleost fish ON bipolar cells (Grant and Dowling, 1995).

In mammals and fish, EAAT5 is found on the synaptic terminals of ON bipolar cells, which depolarize to increments of light (Palmer et al., 2003; Veruki et al., 2006; Wersinger et al., 2006; Ichinose and Lukasiewicz, 2012; Bligard et al., 2020). In mammals, EAAT5 is only found on the synaptic terminals of rod bipolar cells, but not on the terminals of cone bipolar cells at the inner plexiform layer (Veruki et al., 2006; Wersinger et al., 2006; Ichinose and Lukasiewicz, 2012; Bligard et al., 2020). Thus, EAAT5 may modulate the rod signaling pathway in mammalian retina, but not the cone signaling pathways.

## EAAT5'S Functional Role in ROD and Cone Photoreceptors

Does EAAT5 signaling play a functional role in the retina? In the outer retina, EAAT5 is found in the outer plexiform layer on both rod and cone photoreceptor terminals (Picaud et al., 1995b; Grant and Werblin, 1996). Glutamate that is released from rod and cone photoreceptors activates that EAAT5, negatively feeding back, activating a chloride conductance, inhibiting the photoreceptor and reducing the further release of glutamate. In these cases (primarily in lower vertebrates), ECl is more negative than the photoreceptor membrane potential. Thus, activation of the EAAT5 gated chloride conductance results in a hyperpolarization of the photoreceptor and a reduction in the rate of release of glutamate. Rabl et al. (2003) show that activation of EAATs on rods reduces calcium currents, which in turn reduces glutamate release. Postsynaptic partners of photoreceptors, such as off bipolar cells and horizontal cells, are depolarized by glutamate. Activation of the EAAT5 chloride conductance would limit the depolarization in these postsynaptic, retinal neurons. In some mammals, ECl is more positive than the cone resting potential. Thus, EAAT5 activation results in a depolarization and an increase in release of glutamate. Szmajda and Devries (2011) showed that glutamate can spill over and activate neighboring cones, causing them to depolarize when EAAT5 is activated on neighboring cones. The depolarization of neighboring cones by the activation of EAAT5 may act to prime them, allowing them to respond more effectively to darkness (cones are depolarized in the dark and hyperpolarized by light).

## EAAT5 Role on Fish Bipolar Cell Dendrites

EAAT5 is also found post-synaptically on white perch ON bipolar cell dendrites (Grant and Dowling, 1995) (Figure 1). Note that these dendritically localized transporter gated anion channels have not been found in all vertebrate retinas, primarily only fish. ON bipolar cell dendrites contact photoreceptors. Thus, changes in the release of glutamate affect glutamate responses that are mediated by ON bipolar cell dendrites. Grant and Dowling (1995) reported that the cone inputs, but not the rod inputs, were mediated by a glutamate-activated chloride conductance. Pharmacological evidence indicated that this conductance had the properties of a glutamate transporter. It was not affected by ligands that typically activate or block known glutamate receptors. It was likely an EAAT5 glutamate transporter because it exhibited a chloride conductance and was sensitive to glutamate transporter-specific ligands. The rod input, by contrast, was mediated by the more conventional mGluR6 receptors (as in other vertebrate retinas). Glutamate binding to the mGluR6 receptor closed a non-specific cation conductance that is mediated by TRPM1 channels (Koike et al., 2009; Morgans et al., 2009, 2010), hyperpolarizing the bipolar cell (Slaughter and Miller, 1981). These fish neurons are mixed bipolar cells that receive inputs from both rod and cones. The rod input (increased glutamate release in the dark) results in the glutamate activation of mGluR6 receptors, the closing of a non-specific cation TRPM1 channel, and a hyperpolarization of the ON bipolar cell. By contrast, the cone input results in glutamate activation of transporter-mediated chloride conductance and the hyperpolarization of the white perch, ON bipolar cell (Grant and Dowling, 1995). Thus, rod and cone inputs to the carp ON bipolar cell both hyperpolarize the ON bipolar cell in darkness, but they do so by distinct functional mechanisms.

Similar results have been reported for mouse ON bipolar cell dendrites (Tse et al., 2014). Tse et al. (2014) claim that a component of the mouse ON bipolar cell response is mediated by an EAAT-like conductance, as in fish. However, when many components of the mGluR6 and TRPM1 complex are eliminated, they have no ON bipolar responses (Dryja et al., 2005; Gregg et al., 2007; Koike et al., 2009; Morgans et al., 2009). This would suggest that the transporter conductance described by Tse et al. (2014) plays little or no role in ON bipolar cell signaling in mouse. The role of an EAAT conductance on mouse on bipolar cells remains unclear.

## EAAT5'S Function Role At the Inner Plexiform Layer

EAAT5 is also located on the synaptic terminals of fish bipolar cells and mammalian rod bipolar cells, the outputs of bipolar cells, which contact amacrine and ganglion cells (Figure 1). Palmer et al. (2003) showed that EAATs are present at fish ON bipolar cells. They found that EAAT activation elicited a current in isolated, fish synaptic terminals. The authors showed that the pharmacology of the anion current was consistent with an EAAT and not conventional glutamate, GABA, or glycine receptors. The magnitude of the EAAT anion current correlated with glutamate release (glutamate activates the anion conductance), which was assessed with capacitance measurements (glutamate vesicle fusion resulted in capacitance increases).

EAAT5 transporters are found not only on the bipolar cell terminals of fish ON bipolar cells but also on retinal bipolar cells of mammals. Wersinger et al. (2006) showed that EAAT5 transporters are present on the terminals of rat, rod bipolar cells. This special type of ON bipolar cell mediates rod signaling, relaying rod photoreceptor signals to AII or rod amacrine cells. Depolarization of the rod bipolar cells elicited glutamate release and activated EAAT5 transporters. This chloride current, was inhibitory and novel. As with some photoreceptors (see above), activation of EAAT5 causes negative feedback in rod bipolar cells, reducing the further release of glutamate by activating a chloride conductance and reducing excitatory signaling. This current was not affected by GABA or glycine receptor antagonists but was reduced by the glutamate transporter blocker TBOA. Thus, as with teleost fish, this was a novel, inhibitory chloride current that was not mediated by a conventional inhibitory neurotransmitter, such as GABA or glycine, which have been shown to mediate amacrine cell inhibition to bipolar cells (Chavez et al., 2006, 2010; Eggers and Lukasiewicz, 2006; Chavez and Diamond, 2008). Instead, as in fish, this new form of inhibition was presumably mediated by an EAAT5 transporter.

Around the same time as the Wersinger et al. (2006) and Veruki et al. (2006) also showed in rat retina that glutamate transporters mediated a novel inhibition, which was independent of GABA and glycine. Using dual recordings, they showed that glutamate release spilled over and activated neighboring rod bipolar cells. Additional experiments show that the transporter-mediated inhibition affected transmission from the rod bipolar cell to postsynaptic targets. Thus, transporter-mediated inhibition, like conventional inhibition, reduced signaling to postsynaptic neurons. So, transporter-mediated anion conductances modulate retinal signaling, in addition to conventional inhibitory neurotransmitters, such as GABA.

## Transporters Mediate Light-Evoked Signaling in Retina

Light-evoked signaling is also affected by transporter-mediated inhibition (Ichinose and Lukasiewicz, 2012). As noted by Veruki et al. (2006), spillover occurred between neighboring rod bipolar cells. Two spatially defined types of light-evoked inhibition occurred. Transporter-mediated inhibition is spatially restricted to spillover between nearby bipolar cells (~100 microns). By contrast, conventional inhibition, mediated by GABA and glycine, was mediated by amacrine cells and had a larger spatial extent (>500 microns). Activation of the transporter with puffs of D-aspartate reduced the light-evoked bipolar responses and blockade of the transporter current with TBOA enhanced the light-evoked bipolar response (Ichinose and Lukasiewicz, 2012). Note that TBOA (threo-beta-benyloxyaspartate) is a general blocker of EAATs (Shimamoto et al., 1998). A specific EAAT5 blocker does not exist, as noted above.

Blockade of transporter currents with TBOA enhanced exocytosis. Activation of the transporter currents with D-aspartate reduced exocytosis. Taken together, light-evoked signaling from rod bipolar cells is modulated by transporter-mediated inhibition. Thus, the light-evoked output of rod bipolar cells is affected not only by conventional inhibition from GABAergic and glycinergic amacrine cells but also by inhibition caused by the activation of EAAT5. The extent of light-evoked inhibition attributed to transporters is relatively local (<100 microns) and caused by glutamate spillover from neighboring rod bipolar cells. Conventional inhibition, by contrast, occurs over a wider lateral extent (~500 microns) and is mediated by wide field amacrine cells.

Rod bipolar cells receive two types of conventional GABA-mediated inhibition, reciprocal, feedback inhibition (with a narrow spatial extent) and wide-field, lateral inhibition (with a wide spatial extent) (Chavez et al., 2006, 2010). Transporters also mediate a form of feedback inhibition; glutamate released from rod bipolar cells feeds back and activates EAAT5 transporters, which inhibit the rod bipolar cell (Palmer et al., 2003; Veruki et al., 2006; Wersinger et al., 2006; Ichinose and Lukasiewicz, 2012). As with photoreceptors, described above, activation of the glutamate transporter limits the release of additional glutamate by transporter-mediated inhibition. Most of the feedback to rod bipolar cells was mediated by transporter (Bligard et al., 2020). By contrast, all of the wide-field lateral inhibition was mediated by GABA, released from amacrine cell contacts with rod bipolar cell (Eggers and Lukasiewicz, 2006; Chavez et al., 2010; Ichinose and Lukasiewicz, 2012; Bligard et al., 2020). Why do two forms of feedback inhibition exist at rod bipolar cells? It turns out that the two forms of feedback inhibition have distinct time courses. Conventional, GABAergic feedback is fast (both onset and offset) and transporter-mediated feedback is slow (both onset and offset).

*In silico* modeling by Bligard et al. (2020) showed that both the fast, GABA-mediated feedback and the slow transporter-mediated feedback were so that the transmitter release from the rod bipolar cell was optimal (not too large or too little). Either form of feedback alone could suffice to keep transmitter release optimal, but the amount of either form of feedback need to be increased many fold to attain this. The two forms of feedback, with different time courses, complemented each other and were necessary for optimal transmitter release from the rod bipolar cell. Thus, the combination of the quick and transient GABA feedback with the slower and more sustained transporter feedback were optimal for modulating both the initial and prolonged phases of transmit release from rod bipolar cells.

The EAATS, especially EAAT5 (Arriza et al., 1997) and EAAT4 (Fairman et al., 1995), act as an anion channel that can be gated by glutamate. In the retina, EAAT5 is the predominant transporter with anion channel properties (Arriza et al., 1997; Eliasof et al., 1998). EAAT5 is found on photoreceptors and rod bipolar cells (Wersinger et al., 2006) where it can be activated by glutamate that is released from these neurons (Picaud et al., 1995b; Grant and Werblin, 1996; Veruki et al., 2006; Wersinger et al., 2006; Ichinose and Lukasiewicz, 2012; Bligard et al., 2020). The EAAT5 transporter can be activated by light and, when activated, can affect visual processing in the retina (Ichinose and Lukasiewicz, 2012; Bligard et al., 2020).

Works by Gameiro et al. (2011) and Schneider et al. (2014) heterologously expressed EAAT5 and found that, in contrast to Veruki et al. (2006), the transporter-mediated chloride current was smaller and slower. The findings using heterologous expression systems suggest that EAAT5 does not mediate chloride currents in retina. It is unclear why the properties of native and heterologously expressed EAAT5 are different. Native and heterologously expressed behaviors are not always identical. This is the case with heterologously expressed and native neurotransmitter receptors (Parker et al., 2003). It is possible that an intracellular or an extracellular factor present in the native case is not present in the expressed case, accounting for the differences.

## Summary

EAAT5 is found in most vertebrate retinas. Functional studies have been performed on amphibian, teleost fish, and mammals. EAAT5 was initially cloned from the human retina (Arriza et al., 1997). EAAT5 gates an anion conductance (Arriza et al., 1997). This transporter-gated conductance shapes the release of glutamate from photoreceptors and bipolar cells. This form of inhibition (in most cases) modulates visual processing in both the first and second synaptic layers of the retina. Because the signal that gates the transporter-mediated conductance is spatially limited (typically to spillover transmission to nearby neurons), this form of inhibition is typically limited to local feedback. GABA and other conventional neurotransmitters mediate longer range-wide field inhibition (Eggers and Lukasiewicz, 2006; Chavez and Diamond, 2008; Chavez et al., 2010). Bipolar cells receive two types of feedback inhibition at their terminals (Wersinger et al., 2006; Bligard et al., 2020). The rapid, transient feedback that is mediated by synaptic GABA is complemented by the slow, sustained feedback inhibition that is mediated by EAAT5 to optimally control signaling between mammalian rod bipolar cells and their postsynaptic targets.

There is still some uncertainty about the native anion channel gated by glutamate's interaction with EAATs. Most of the literature suggests that EAAT5 is the native anion channel gated by glutamate (Arriza et al., 1997; Eliasof et al., 1998). However, results from heterologously expressed EAAT5 show functional differences (Gameiro et al., 2011; Schneider et al., 2014). The differences in EAAT5 function could be attributed to processes in the native tissue that are not present in expression systems. Boehmer et al. (2005) show that EAAT5 function may be regulated by kinases. Additional work is needed in the regulation of EAATS in the retina.

It is also possible that another EAAT contributes to retinal processing. This is suggested by the functional differences between heterologously expressed EAAT5 (Gameiro et al., 2011) and the putative native EAAT5 (Veruki et al., 2006). It is still not fully understood what the roles of EAATs are in visual processing.

## Author Contributions

All authors contributed to manuscript, read, and approved the submitted version.

## Conflict of Interest

The authors declare that the research was conducted in the absence of any commercial or financial relationships that could be construed as a potential conflict of interest.
